# Accuracy and residual volume of intravitreal injection syringes in administration of very small volumes

**DOI:** 10.1038/s41433-023-02670-1

**Published:** 2023-07-27

**Authors:** Gustavo Barreto Melo, Lucas Andrade Santos, Lydianne Lumack do Monte Agra, Carsten Meyer, Morten Carstens Moe, Øystein Kalsnes Jørstad

**Affiliations:** 1 Hospital de Olhos de Sergipe, Aracaju, SE Brazil; 2 Federal University of São Paulo, São Paulo, SP Brazil; 3 Federal University of Sergipe, Lagarto, Brazil; 4 Philipps University of Marburg, Marburg, Germany; 5 Department of Ophthalmology, Oslo University Hospital and University of Oslo, Oslo, Norway

**Keywords:** Drug therapy, Adverse effects

## Introduction

Intravitreal injections (IVIs) are widely used to deliver therapeutic agents for various retinal conditions. The syringe accuracy is a critical factor in achieving optimal treatment outcomes and minimizing potential risks in IVIs, such as unpredictable variation in drug volume (and thereby therapeutic effect) and intraocular pressure (IOP) rise [1–5]. Smaller volumes than the typical 0.05 mL, albeit harder to deliver with common 1-mL syringes, could mitigate the impact on IOP and drug wastage.

In this study, we evaluated the accuracy and residual volume of three syringe models that can potentially administer very small volumes intravitreally.

## Materials and methods

**Fig. 1:**
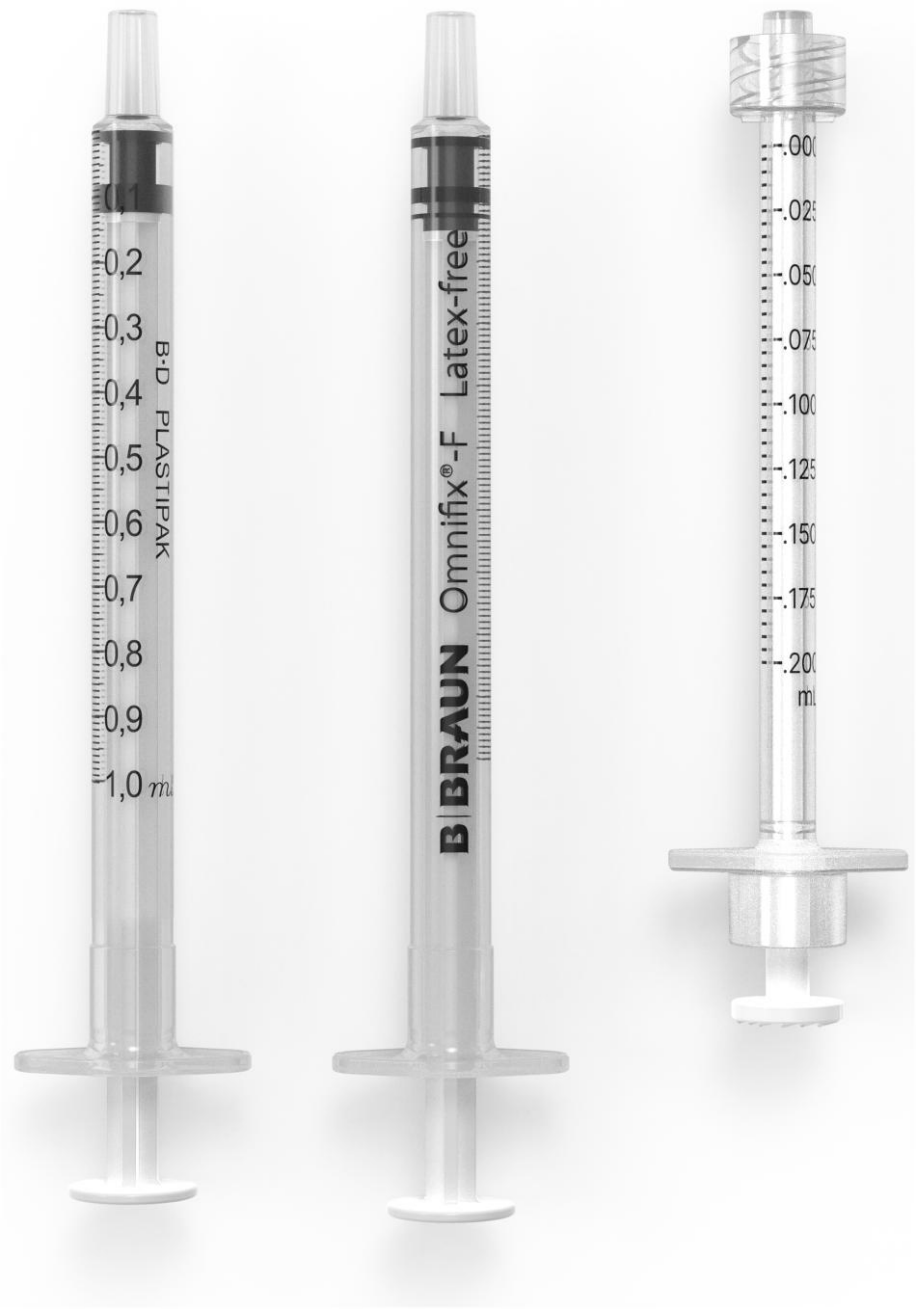
The syringes tested in the study. Left: Omnifix-F from B. Braun Medical Inc. Middle: BD Plastipak from Becton Dickinson S.A. Right: Zero Residual EDT from SJJ Solutions BV.

We investigated three syringe models (Fig. 1): BD Plastipak 1 mL luer slip (Becton Dickinson S.A., Madrid, Spain), Omnifix-F 1 mL (B. Braun Medical Inc., Melsungen, Germany), and Zero Residual Enhanced Dosing Technology (EDT) 0.2 mL (SJJ Solutions BV, Den Haag, Netherlands). Notably, the last model is designed to deliver very small volumes accurately. The BD Plastipak and the Omnifix-F were tested with a standard 30-gauge BD PrecisionGlide needle (Becton Dickinson S.A., Curitiba, Brazil), whereas the Zero Residual EDT was assembled with a 30-gauge Zero Residual needle (SJJ Solutions BV, Den Haag, Netherlands).

A trained member of the research team prepared and assessed each syringe-needle setup. Distilled water was used as the injection fluid, and volumes of 10, 20, 25, and 50 µL were tested. The weight of the syringe-needle setup was measured before and after liquid aspiration and after ejection. The extra syringe-needle weight after liquid ejection defined the residual volume. Each simulation utilized a new syringe and needle. The weight was measured with an analytical balance (OHAUS Model PR224, Parsippany, NJ). The study was conducted at a stable 23 °C room temperature and 70% humidity.

## Results

**Fig. 2:**
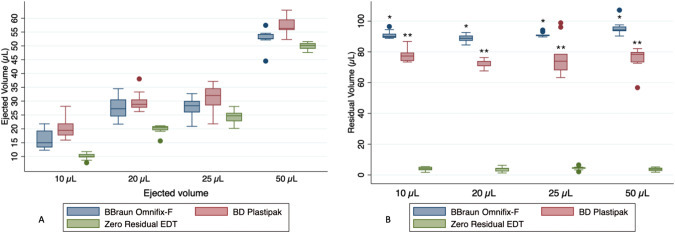
Accuracy and residual volume of the tested syringes. **A** illustrates the variation for different ejection volumes (10, 20, 25, and 50 µL). *p < 0.001 in comparison to Zero Residual EDT. **B** shows the residual volumes after ejection. *p < 0.001 in comparison to both Zero Residual EDT and BD Plastipak; **p < 0.001 in comparison to both Zero Residual EDT and Omnifix-F.

The Zero Residual EDT syringe demonstrated significantly lower volumes delivered and closer to the target volume across all tested volumes compared to the BD Plastipak syringe (*p* < 0.001), as shown in Fig. 2A. In the comparison between the Zero Residual EDT and the B. Braun Omnifix-F syringe, significant differences were observed at volumes of 10 and 20 µL. Furthermore, a statistically significant difference in residual volume was found between the three syringes for all volumes, with the Zero Residual EDT syringe exhibiting the lowest residual volume (Fig. 2B).

## Discussion

The findings of this study highlight the importance of both syringe accuracy and residual volume in the setting of the very small volume delivered during IVIs. The Zero Residual EDT syringe performed better than the BD Plastipak and Omnifix-F syringes with regard to both accuracy and residual volume. The ability to accurately administrate very small volumes is a prerequisite for developing intraocular drugs with lower risk of increased IOP, which, for instance, has been associated with current pre-filled aflibercept syringes [4, 5]. Moreover, minimizing the residual volume of the syringe-needle setup can lead to considerable cost savings by reducing medicine waste. Further research and development in the field of syringe design for IVIs are warranted to explore the full potential of achieving accurate drug delivery, thereby optimizing the benefits and minimizing the risks of this standard ophthalmic procedure.

## Data Availability

Additional data will be made available upon reasonable request.

## References

[1] Melo GB, Cruz NFSD, Emerson GG, Rezende FA, Meyer CH, Uchiyama S (2021). Critical analysis of techniques and materials used in devices, syringes, and needles used for intravitreal injections. Prog Retin Eye Res.

[2] Agra LLM, Sverstad A, Chagas TA, Araújo RH, Oliveira LG, Kristianslund O (2023). Accuracy, precision, and residual volume of commonly used syringes for intravitreal injections and the impact on intraocular pressure. Ophthalmol Ret.

[3] Meyer CH, Liu Z, Brinkmann C, Rodrigues EB, Helb HM (2012). Accuracy, precision and repeatability in preparing the intravitreal dose with a 1.0-cc syringe. Acta Ophthalmol.

[4] Guest JM, Malbin B, Abrams G, Parendo A, Das S, Okeagu C (2022). Accuracy of intravitreal injection volume for aflibercept pre-filled syringe and BD Luer-Lok one-milliliter syringe. Int J Retin Vitreous.

[5] Gallagher K, Raghuram AR, Williams GS, Davies N (2021). Pre-filled aflibercept syringes-variability in expressed fluid volumes and a case series of transient central retinal artery occlusions. Eye.

